# Research hotspots and frontiers of post-stroke aphasia rehabilitation: a bibliometric study and visualization analysis

**DOI:** 10.3389/fnhum.2023.1176923

**Published:** 2023-05-11

**Authors:** Huan Wang, Ziping Cai, Shengjuan Li, Jiaxing Zheng, Yuyao Xie, Yuanyuan He, Chen Li, Dongxiang Zheng

**Affiliations:** ^1^College of Nursing, Jinan University, Guangzhou, China; ^2^Department of Neurology and Stroke Center, The First Affiliated Hospital, Jinan University, Guangzhou, China; ^3^College of Rehabilitation, Jinan University, Guangzhou, China

**Keywords:** post-stroke aphasia, rehabilitation, bibliometric analysis, VOSviewer, CiteSpace

## Abstract

**Background:**

Aphasia is a common complication of stroke and is associated with high morbidity and mortality rates. Rehabilitation plays a crucial role in the comprehensive management of post-stroke aphasia and its consequences. However, bibliometric analysis in the field of post-stroke aphasia rehabilitation is still lacking. This study aimed to comprehensively identify assistance networks, analyze research trends, focus on hot and cutting-edge health topics related to post-stroke aphasia rehabilitation, and inform future research guidelines.

**Methods:**

The Web of Science Core Collection (WoSCC) electronic database was searched from inception to January 4, 2023 to identify studies related to post-stroke aphasia rehabilitation. Bibliometric analysis and visualization of country, institution, journal, author, reference, and keywords were performed using CiteSpace and VOSviewer software.

**Results:**

A total of 2,325 papers were included in the analysis, with a progressive increase in the number of articles published each year. The USA was the country with the most publications (809 articles), and the University of Queensland was the institution with the most publications (137 articles). The subject area of post-stroke aphasia rehabilitation is dominated by clinical neurology (882 articles). Aphasiology was the journal with the most publications (254 articles) and the most cited journal (6,893 citations). Worrall L was the most prolific author (51 publications), and Frideriksson J was the most cited author (804 citations).

**Conclusion:**

By using bibliometrics, we provided a comprehensive review of studies related to post-stroke aphasia rehabilitation. Future research hotspots on topics related to post-stroke aphasia rehabilitation will mainly focus on the plasticity mechanisms of neurolinguistics networks, language function assessment, language rehabilitation modalities, and patients’ rehabilitation needs and participation experiences in post-stroke aphasia. This paper provides systematic information that is worth exploring in the future.

## 1. Introduction

The 2019 Global Burden of Disease Study revealed that stroke is the second leading cause of death and the third leading cause of disability worldwide (Feigin et al., 2021). Furthermore, more than one-third of stroke patients experience aphasic symptoms (Flowers et al., 2016). Post-stroke aphasia (PSA) is an acquired language disorder caused by brain damage that can lead to difficulties with comprehension, speech production, and literacy and is one of the most serious consequences of stroke (Picano et al., 2021). Studies have confirmed that patients with post-stroke aphasia have higher risks of death and psychological problems, such as anxiety and depression, than non-aphasic stroke patients (Baker et al., 2018; Baker et al., 2020). However, surprisingly, post-stroke aphasia is often overlooked in the community, and gaps in diagnosis and treatment prevent patients from receiving the rehabilitation support they need (Wray and Clarke, 2017). In addition, many people with post-stroke aphasia experience social isolation due to ongoing communication difficulties, and they often protect themselves by avoiding social interaction; thus, these individuals can have difficult family relationships and a lower risk of returning to work, thereby reducing their quality of life (Dalemans et al., 2010).

Based on the reorganization mechanisms of the brain’s neural networks, current research suggests that a key factor in positive language outcomes is the provision of several hours of intensive language training per week (Brady et al., 2016). In addition, pharmacology (Floel and Cohen, 2010) and non-invasive brain stimulation, such as transcranial direct current stimulation (tDCS), as a substitute or adjunctive therapy for traditional rehabilitation methods can help maximize the language recovery process (Picano et al., 2021).

However, although an increasing number of scholars are focusing on the rehabilitation of post-stroke aphasia, the direction and hotspots of research in this field are still unclear. No comprehensive bibliometric analysis has examined the rehabilitation of post-stroke aphasia. A bibliometric visual analysis is a quantitative analysis that integrates numerical and statistical methods to describe the data and patterns collected in a specific research area by using numerical or other estimation techniques (Ellegaard and Wallin, 2015) and then visualizing the collected data using computer graphics and image processing techniques that can be visualized (Chen, 2004). To determine the fundamental situation and development trend of post-stroke aphasia rehabilitation, this study provides a statistical analysis of the published literature in the field. This study also provides visual information and research directions for scholars to guide future research.

## 2. Materials and methods

### 2.1. Data source and publication search strategy

**FIGURE 1:**
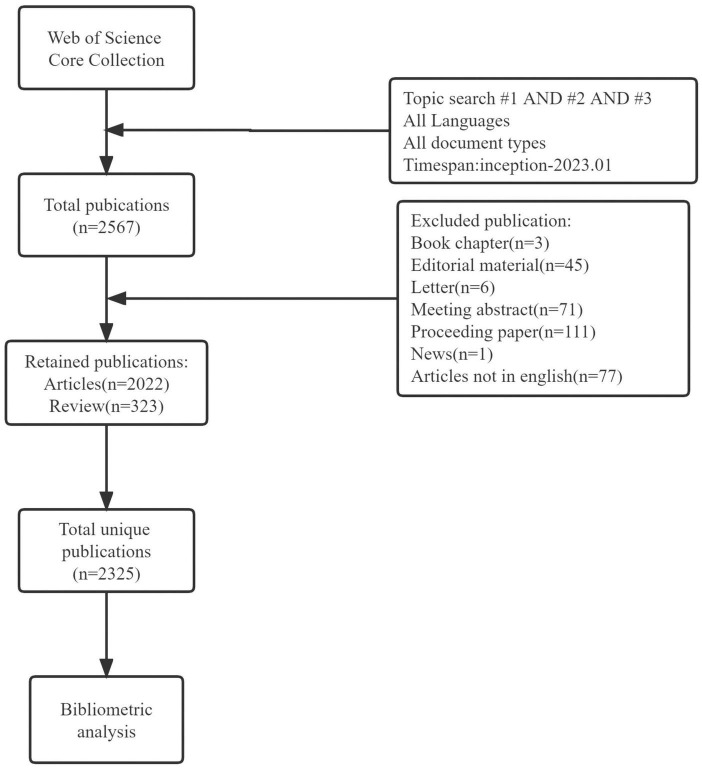
Shows the flowchart of study identification and selection. Topic search #1=(stroke OR apoplexy OR “cerebrovascular accident” OR “brain vascular accident” OR “cerebral hemorrhage” OR encephalorrhagia OR “cerebral ischemia”); topic search #2=(aphasia OR logasthenia OR logagnosia OR logamnesia OR alogia OR anepia OR “acquired aphasia” OR dysphasia); Topic search #3=(rehabilitation OR recovery).

In this study, the Web of Science Core Collection (WoSCC) was selected as the source database for data retrieval. To eliminate bias caused by updating the database, we systematically searched all publications published from inception to January 4, 2023, within 1 day. The data retrieval strategy was as follows: TS = (stroke OR apoplexy OR “cerebrovascular accident” OR “brain vascular accident” OR “cerebral hemorrhage” OR “encephalorrhagia” OR “cerebral ischemia”) AND TS = (aphasia OR logasthenia OR logagnosia OR logamnesia OR alogia OR anepia OR “acquired aphasia” OR dysphasia) AND TS = (rehabilitation OR recovery). The type of literature was limited to articles and reviews, and the titles and abstracts of all publications retrieved by the above search strategy were screened. To ensure the accuracy of the bibliometric analysis, we excluded irrelevant literature, including book chapters, editorial materials, conference abstracts, etc. To facilitate further literature content analysis, we limited the publication language type to English and then removed duplicates, resulting in a total of 2,325 original articles (including articles and reviews) published in English. Figure 1 illustrates the process of study identification and selection. The complete record of each article, including country/institution, journal, authors, references, and keywords, was downloaded from the WoSCC database in text format.

### 2.2. Bibliometric analysis software tools

An analysis of the research lineage, status, trends, and hotspots in the rehabilitation of post-stroke aphasia was carried out using VOSviewer 1.6.18 and CiteSpace 6.1.R6. VOSviewer is a scientific measurement application for generating and viewing bibliometric maps (Van Eck and Waltman, 2010). This study used VOSviewer to obtain networks of collaboration (country, institution, and co-authorship networks), networks of influence (co-authors, co-journal, and co-reference networks), and keyword co-occurrence analysis, where the node size of a network represents its number or frequency and the connections between nodes represent their collaborative relationships (e.g., co-occurrence or co-citation).

CiteSpace can combine bibliometric analysis and systematic mapping by using visual analysis methods and data mining algorithms to capture hot spots and research trends to explore research areas (Chen, 2006), also known as “scientific knowledge mapping” (Synnestvedt et al., 2005). This study uses CiteSpace to examine the dual-map overlap of journals and the cluster view and burst detection of cited literature. The dual-map overlap of journals is a new way of displaying the distribution of articles and citation trajectories across disciplines, providing an understanding of interdisciplinary relationships in the field (Li et al., 2022). For reference analysis, a log-likelihood ratio (LLR) strategy was used, with the type “keyword” selected, to generate co-citation clusters of references. Burst detection was used to summarize the top 20 papers with the highest citation frequency and the strongest citation burst. Three different structural measures, i.e., mediated centrality, modularity, and weighted average profile value, are used to indicate the quality of the clusters (Chen et al., 2010). Mediated centrality is an important measure of a node’s position in the network by computing the shortest path between all node pairs (Chen et al., 2009). It is shown that nodes with high intermediary centrality can identify boundary spanning potentials that may lead to transformative discoveries (Chen, 2014, 2017). The modularity value (Q-value) is a measure of the degree to which the network is classified as modular, with Q-values ranging between (0, 1), and >0.3 is considered acceptable (Newman, 2006). The weighted average profile value (S-value) is used to estimate the degree of uncertainty in explaining the nature of the network or individual clusters, with S-values ranging between (0, 1), and >0.7 is considered acceptable for homogeneity (Rousseeuw, 1987; Sabe et al., 2023). For keyword analysis, the top 20 keywords were summarized, and the top 25 keywords with the strongest citation burst were charted. In addition, the number of publications published per year was analyzed using the exponential growth function in Excel.

## 3. Results

### 3.1. Analysis of publication outputs

**FIGURE 2:**
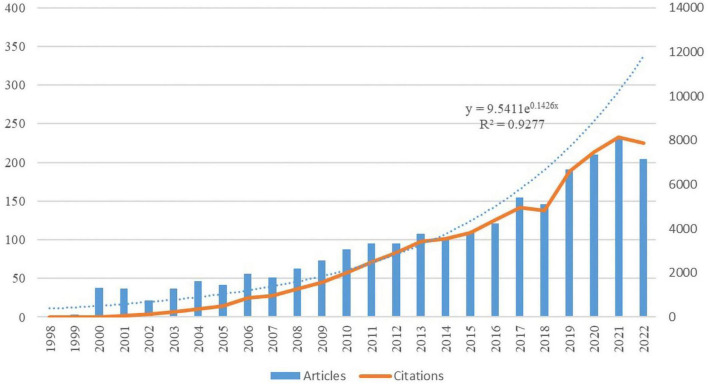
Annual publication trend of post-stroke aphasia rehabilitation.

A total of 2,325 papers were included in the analysis, including 2022 articles and 323 reviews published between 1998 and 2022. The total h-index is 111, and the cumulative number of citations is 68,471, with an average of 29.45 citations per article. Figure 2 shows the annual number of publications and citations for rehabilitation of patients with aphasia after stroke, with a general trend of continuous but erratic growth. The number of publications in this field began to increase slowly from 1998 to 2007, accelerated starting in 2008, and peaked during 2019–2022, indicating that rehabilitation of patients with aphasia after stroke received greater attention during this period. The exponential growth model assessing the relationship between annual volume and year of publication showed that the model matched the trend in annual volume (R^2^ = 0.9277).

### 3.2. Country/region and institution contributions

**FIGURE 3:**
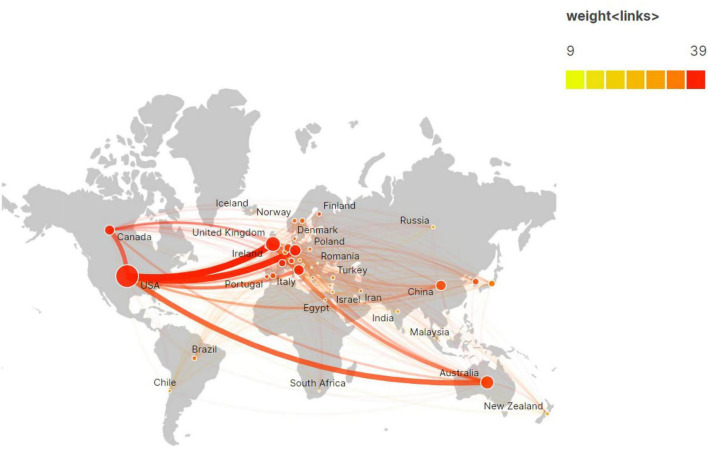
Network of international cooperation of post-stroke aphasia rehabilitation in geographic visualization.

Based on the authors’ addresses, studies on post-stroke aphasia rehabilitation came from 72 countries/regions and 2,575 institutions. The top 10 countries and institutions in terms of the number of publications are shown in Table 1. The USA ranked first for the number of publications and citations in the field (809 articles, 28,297 citations), followed by the United Kingdom (343 articles, 14,352 citations), Australia (273 articles, 7,917 citations), Germany (205 articles, 9,022 citations), and Italy (171 articles, 6,153 citations); these five countries published 77.46% of the total number of articles. The network of cooperation between countries is shown in Figure 3, where the size of the nodes indicates the volume of publications produced in each country. The thickness and color shades of the connecting lines reflect the intensity of cooperation among countries or regions, and the closer the color of the nodes is to red, the greater the intensity of their research cooperation. The results show that the USA has the highest international cooperation strength (with 386 link strength), and the frequent cooperation among the USA, UK, Germany, and Australia has formed a wide research cooperation network.

**TABLE 1:** Ranking of top 10 countries and institutions involved in the post-stroke aphasia rehabilitation field.

Rank	Country/Region	Count (%)	Citations	Institution	Count (%)	Citations
1	USA	809 (34.80%)	28,297	Univ Queensland	137 (5.89%)	3,771
2	United Kingdom	343 (14.75%)	14,352	Johns Hopkins Univ	85 (3.66%)	2,363
3	Australia	273 (11.74%)	7,917	Boston Univ	64 (2.75%)	1,775
4	Germany	205 (8.82%)	9,022	UCL	60 (2.58%)	2,026
5	Italy	171 (7.35%)	6,153	Northwestern Univ	57 (2.45%)	1,456
6	China	169 (7.27%)	1,747	Edith Cowan Univ	56 (2.41%)	1,304
7	Canada	157 (6.75%)	7,405	La Tobe Univ	55 (2.37%)	2,461
8	Netherlands	107 (4.60%)	4,777	Univ Toronto	44 (1.89%)	2,644
9	France	82 (3.53%)	3,107	Univ Montreal	43 (1.85%)	1,652
10	Japan	68 (2.92%)	857	City Univ London	42 (1.81%)	1,587

**FIGURE 4:**
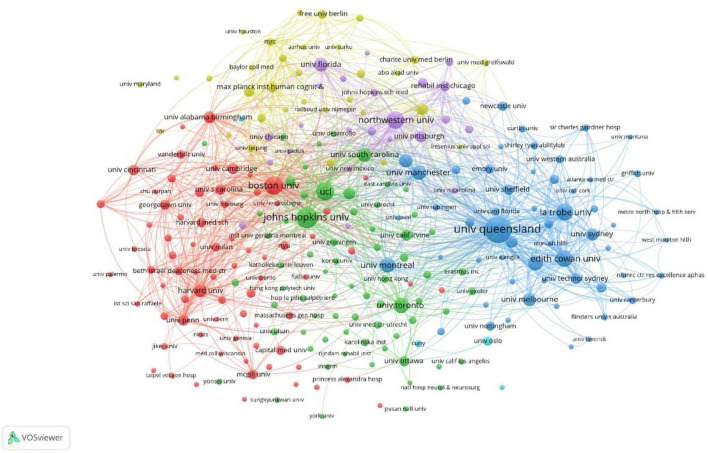
Institutional cooperation network diagram of post-stroke aphasia rehabilitation.

The institutions with the highest number of publications and citations are the University of Queensland (137 articles, 3,771 citations), followed by Johns Hopkins University (85 articles, 2,363 citations), Boston University (64 articles, 1,775 citations), University College London (60 articles, 2,026 citations), and Northwestern University (57 articles, 1,456 citations). The collaborative networks among institutions are shown in Figure 4. A total of 257 institutions were identified as having published a minimum of five articles, forming six color clusters based on the intensity of collaboration. There were extensive research collaboration networks among the clusters, with red indicating the cluster containing the most institutions (73 institutions).

### 3.3. Research categories

**FIGURE 5:**
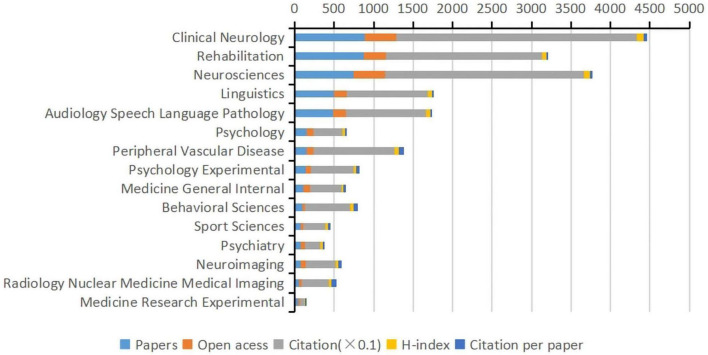
The top 15 subject categories of Web of Science in terms of articles, open-access articles, citations, H-index, and citations per article (WoS).

The 2,325 publications were grouped into 90 WoS subject categories. Figure 5 shows the top 15 categories. Among the top 15 subject categories, clinical neurology had the highest number of articles (882 articles), the highest number of open-access articles (407 articles), the highest number of citations (3,050 citations), and the highest h-index (h-index = 86), followed by rehabilitation (877 articles) and neuroscience (745 articles). Peripheral vascular disease had the highest average number of citations in the literature (66.97 per article).

### 3.4. Journal and co-cited journal distribution

**FIGURE 6:**
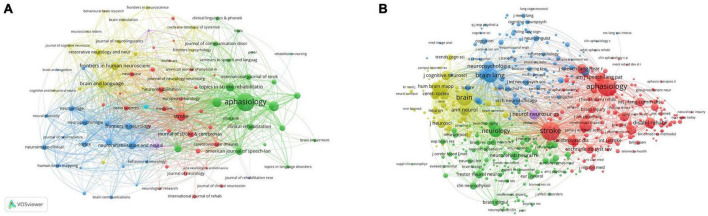
Analysis journals of post-stroke aphasia rehabilitation. **(A)** Journals co-occurrence analysis. **(B)** Co-cited journals analysis.

The papers on post-stroke aphasia rehabilitation were published in 449 academic journals, and the co-occurrence relationship between journals was visualized by VOSviewer, as shown in Figure 6A. Rehabilitation (73 articles, 3.14%), and Stroke (63 articles, 2.17%) had the highest impact factors (IF10.2) among the top 10 journals. According to the visual analysis of co-cited journals, the most cited journals were Aphasiology (6,893 citations), Stroke (6,881 citations) and Brain (4,026 citations). The co-occurrence relationship between co-cited journals is shown in Figure 6B. The top 10 journals and co-cited journals related to post-stroke aphasia rehabilitation are shown in Table 2.

**TABLE 2:** Top 10 journals and co-cited journals of post-stroke aphasia rehabilitation.

Rank	Journal	Count (%)	IF (2022)	JCR	Co-cited journal	Citations	IF (2022)	JCR
1	Aphasiology	254 (10.92%)	1.902	Q2	Aphasiology	6,893	1.902	Q2
2	Disability and Rehabilitation	73 (3.14%)	2.439	Q1	Stroke	6,881	10.170	Q1
3	Stroke	63 (2.71%)	10.170	Q1	Brain	4,026	15.255	Q1
4	Archives of Physical Medicine and Rehabilitation	62 (2.67%)	4.060	Q1	Brain and Language	3,820	2.781	Q1
5	Topics in Stroke Rehabilitation	55 (2.37%)	2.177	Q2	Neuroimage	3,450	7.400	Q1
6	Brain and Language	50 (2.15%)	2.781	Q1	Neurology	2,674	11.800	Q1
7	Frontier in Human Neuroscience	47 (2.02%)	3.473	Q2	Archives of Physical Medicine and Rehabilitation	2,383	4.060	Q1
8	Neurorehabilitation and Neural Repair	44 (1.89%)	4.895	Q1	Annals of Neurology	1,748	11.274	Q1
9	American Journal of Speech-language Pathology	40 (1.72%)	4.018	Q1	Neuropsychologia	1,676	3.054	Q2
10	Neuropsychological Rehabilitation	40 (1.72%)	2.928	Q3	Cortex	1,592	4.644	Q1

**FIGURE 7:**
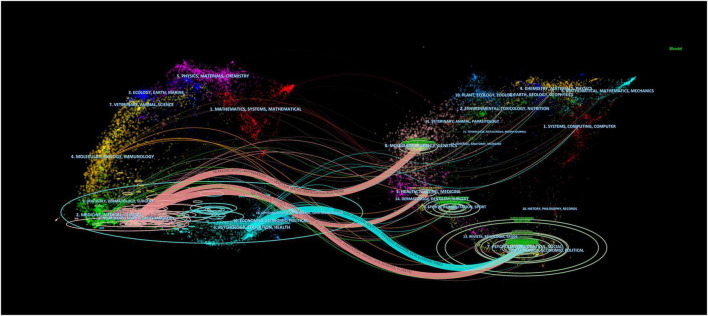
The dual-map overlay of journals in post-stroke aphasia rehabilitation.

The dual-map overlaps of the journals is shown in Figure 7. The map is divided into two parts, with the citing journals distributed on the left and the cited journals on the right. On the left side of the map, the length of the ellipse indicates the number of authors, the width of the ellipse indicates the number of publications, and the curves between the left and right sides of the map are citation links, which show the complete citation context. The Z score function further highlights stronger and smoother trajectories, with higher scores indicated by thicker links. We identified four main citation trajectories (pink and blue), with journals in Medicine, Medical, Neurology, and Sports (pink trajectory) being significantly more heavily cited by Psychology, Education, Social, Economics (Z = 6.21, f = 14,874), Molecular, Biology, Genetics (Z = 3.52, f = 8,769), and Health, Nursing, Medicine (Z = 2.06, f = 5,463) fields. In addition, journals in Psychology, Education, and Health (blue track) were influenced by journals in Psychology, Education, Social, and Economics (Z = 3.89, f = 9,615).

### 3.5. Authors and co-cited authors

**FIGURE 8:**
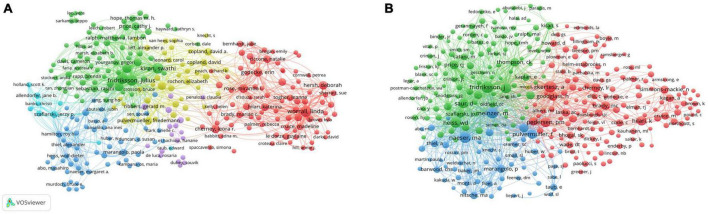
Co-authorship analysis of authors in post-stroke aphasia rehabilitation. **(A)** Network diagram of influential authors. **(B)** Network diagram of influential co-cited authors.

A total of 7,903 authors have contributed to papers on post-stroke aphasia rehabilitation. We can identify the representative scholars and core research strengths in this field by analyzing the authors of the literature. Table 3 displays the top 10 authors and co-cited authors of post-stroke aphasia rehabilitation research. Among the top 10 authors in terms of the number of publications, Worrall L from the University of Queensland has the most publications (51 articles, 2.19%), followed by Fridriksson J (49 articles, 2.11%). Frideriksson J (804 citations) was the most cited author, followed by Kertesz A (772 citations) and Naeser M (674 citations). Figure 8 show the collaboration between authors and co-cited authors in the field, with circles indicating authors, the lines connecting the circles reflect the connections between authors, and the colors indicate clusters of authors involved in the collaboration. In Figure 8A, 284 authors with at least five publications were clustered into six clusters, with closer author collaboration between clusters and less linkage between different clusters, thus suggesting that research teams/labs conducting research related to stroke aphasia rehabilitation should further strengthen cross-regional collaboration in the future.

**TABLE 3:** Top 10 authors and co-cited authors of post-stroke aphasia rehabilitation.

Rank	Author	Count (%)	Co-cited author	Citations
1	Worrall L	51 (2.19%)	Frideriksson J	804
2	Fridriksson J	49 (2.11%)	Kertesz A	772
3	Hillis A E	48 (2.06%)	Naeser M	674
4	Kiran S	47 (2.02%)	Meinzer M	631
5	Rorden C	31 (1.33%)	Hillis A	577
6	Boniha L	31 (1.33%)	Heiss W	539
7	Rose M I	28 (1.20%)	Pedersen P	530
8	Godecke E	27 (1.16%)	Saur D	504
9	Cherney L R	27 (1.16%)	Hilari K	490
10	Hersh D	26 (1.16%)	Pulvermuller F	474

### 3.6. Analysis of research hotspots

#### 3.6.1. Publications with the highest number of citations

**FIGURE 9:**
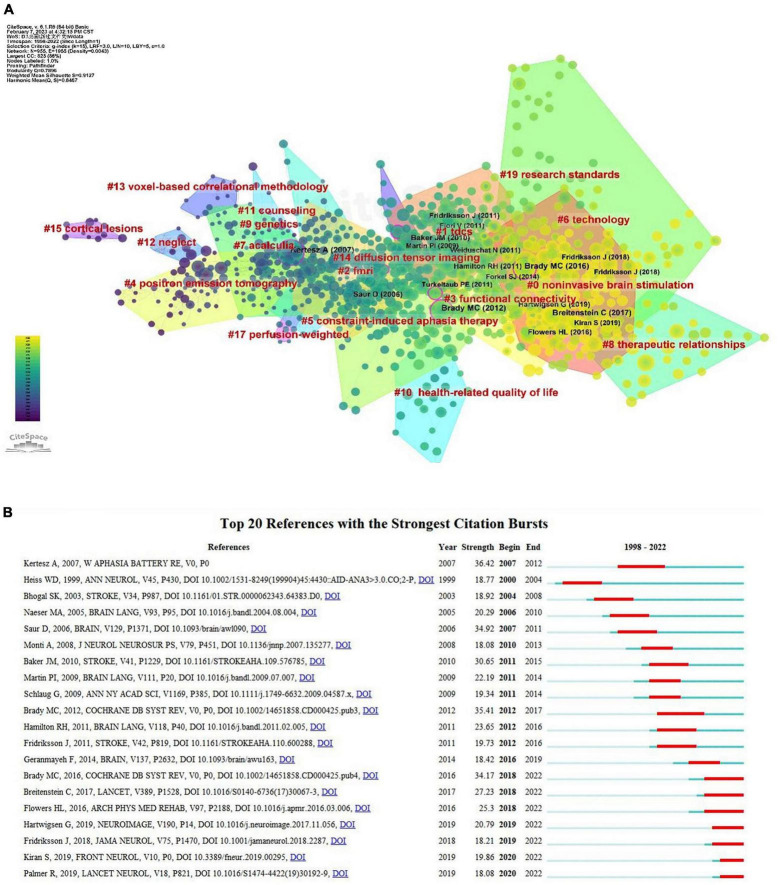
References analysis of post-stroke aphasia rehabilitation. **(A)** Co-citation references clustering. **(B)** Top 20 references with the strongest citation bursts.

Table 4 displays the most cited references from the 2,325 retrieved publications. Speech and language therapy for aphasia following stroke occupied the first place in terms of citations (102), with a mediated centrality of 0.14, from the Cochrane Database of Systematic Reviews (IF12.008, Q1). Using the clustering function of CiteSpace to group co-cited references as shown in Figure 9A, the modularity Q was 0.7896 (>0.5), indicating that the clustering network was reasonable, the S value was 0.9127 (>0.7), indicating that the homogeneity of the clustering network was acceptable. The gradation of the circles from purple to yellow depicts the temporal dimension and can represent a shift in the focus and direction of the study. “Non-invasive brain stimulation” was the largest cluster (cluster #0), followed by “transcranial direct current stimulation (tDCS)” (cluster #1), “fMRI” (cluster #2), and “functional connectivity” (cluster #3); other significant clusters included “positron emission tomography,” “constraint-induced aphasia therapy,” “technology,” “therapeutic relationships,” “genetics,” “health-related quality of life” and others.

**TABLE 4:** Top 10 co-cited references of post-stroke aphasia rehabilitation.

Rank	References	Cited frequency	Centrality	Document title	Source	IF (2022)	JCR
1	Brady et al., 2016	102	0.14	Speech and language therapy for aphasia following stroke	Cochrane Database of Systematic Reviews	12.008	Q1
2	Kertesz, 1982	99	0.18	Western aphasia battery test manual	Psychological Corporation		
3	Breitenstein et al., 2017	85	0.07	Intensive speech and language therapy in patients with chronic aphasia after stroke: a randomized, open-label, blinded-endpoint, controlled trial in a health-care setting	Lancet	202.731	Q1
4	Baker et al., 2010	74	0.03	Using transcranial direct-current stimulation to treat stroke patients with aphasia	Stroke	10.170	Q1
5	Saur et al., 2006	66	0.05	Dynamics of language reorganization after stroke	Brain	15.255	Q1
6	Flowers et al., 2016	63	0.01	Post-stroke aphasia frequency, recovery, and outcomes: a systematic review and meta-analysis	Archives of Physical Medicine and Rehabilitation	4.060	Q1
7	Hamilton et al., 2011	57	0	Mechanisms of aphasia recovery after stroke and the role of non-invasive brain stimulation	Brain and Language	2.781	Q1
8	Hartwigsen and Saur, 2019	57	0.03	Neuroimaging of stroke recovery from aphasia - Insights into plasticity of the human language network	Neuroimage	7.400	Q1
9	Fridriksson et al., 2011	57	0.02	Transcranial direct current stimulation improves naming reaction time in fluent aphasia: a double-blind, sham-controlled study	Stroke	10.170	Q1
10	Fridriksson et al., 2018	56	0.02	Transcranial Direct Current Stimulation vs Sham Stimulation to Treat Aphasia After Stroke: A Randomized Clinical Trial	JAMA Neurology	29.907	Q1

#### 3.6.2. Analysis of reference citation bursts

The most frequently cited literature with the strongest citation bursts is considered the basis for future cutting-edge research, as shown in Figure 9B, which shows the top 20 most cited bursts and is arranged chronologically. The blue line indicates the observed time interval from 1998 to 2022, and the red color represents the duration of the burst, thus illustrating research hotspots and durations. The Western Aphasia Battery-Revised had the highest outbreak citation strength (strength = 36.42) and was an assessment of language function in patients with acquired neurological disorders (e.g., stroke, head injury) with aphasia (Kertesz, 2007). There is a continuing outbreak of citations for seven articles, four of which deal with rehabilitation methods for post-stroke aphasia, including various means of speech and language rehabilitation and transcranial direct current stimulation (tDCS) therapy (Brady et al., 2016; Breitenstein et al., 2017; Fridriksson et al., 2018; Palmer et al., 2019). A previous meta-analysis thoroughly examined the incidence, rehabilitation trends, and outcomes of post-stroke aphasia (Flowers et al., 2016), thus serving as an invaluable resource for understanding post-stroke aphasia for researchers. Two other papers provide insights into the mechanisms of functional recovery and neuroplasticity of language neural networks in post-stroke aphasia (Hartwigsen and Saur, 2019; Kiran and Thompson, 2019).

#### 3.6.3. Clustering analysis and keyword occurrence frequency

**FIGURE 10:**
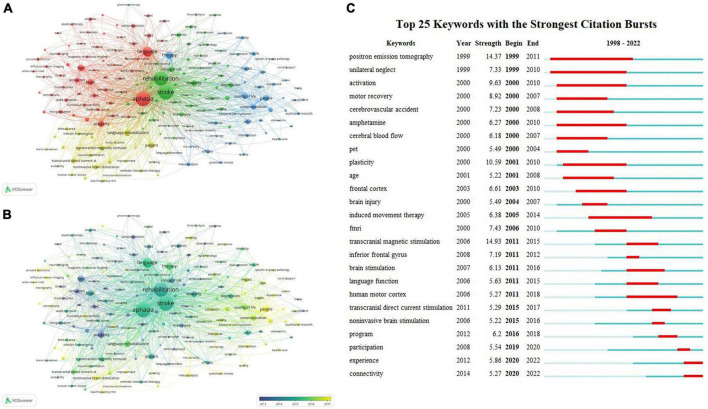
Keywords analysis of post-stroke aphasia rehabilitation. **(A)** Cluster view of keywords. **(B)** Time-overlapping visualization of keywords. **(C)** Top 25 keywords with the strongest citation bursts.

First, keywords with similar meaning were combined to determine the frequency of keyword publication, and the top 20 keywords are summarized as shown in Table 5. The keyword network can show the main research objects and research hotspots, and we use VOSviewer to implement the co-occurrence network of keywords and cluster the keywords into groups. The minimum number of keyword occurrences was set to 20; 172 out of 8,016 keywords met the criteria and were thus included in the analysis and divided into 4 color clusters. The keyword clustering visualization is shown in Figure 10A. In addition to “stroke, aphasia, rehabilitation”, the main keywords in the red cluster included “language, plasticity, fMRI, brain, activation”; the main keywords in the green cluster included “disability, clinical trials, depression, prognosis”; the main keywords in the blue cluster included “people, quality of life, communication, participation”; and the keywords in the yellow cluster included “transcranial magnetic stimulation, tDCS, non-invasive brain stimulation.” In addition, we performed a visualization of the temporal overlap of keywords, as shown in Figure 10B, with the earlier keywords shown in purple and the yellow ones indicating the most recent keywords. A change from purple to yellow represents a shift in research trends and hotspots.

**TABLE 5:** Top 20 keywords of post-stroke aphasia rehabilitation.

Rank	Keyword	Frequency	Total link strength	Rank	Keyword	Frequency	Total link strength
1	stroke	1,599	9,159	11	patient	203	1,308
2	aphasia	1,589	9,635	12	disability	192	1,204
3	rehabilitation	1,438	8,622	13	clinical trial	186	1,143
4	language	630	4,230	14	quality of life	185	1,172
5	therapy	316	2,056	15	communication	162	1,147
6	language rehabilitation	260	1,826	16	transcranial magnetic stimulation	147	1,142
7	fMRI	251	1,751	17	depression	141	884
8	plasticity	242	1,751	18	adult	135	841
9	people	220	1,358	19	transcranial direct current stimulation	121	955
10	brain	208	1,344	20	activation	110	793

#### 3.6.4. Keyword citation burst analysis

Figure 10C displays the top 25 keywords from 1998 to 2022 with the highest citation bursts. In addition to the search term-related terms, the top five keywords with the strongest citation explosion were transcranial magnetic stimulation (bust strength = 14.93), positron emission tomography (bust strength = 14.37), plasticity (bust strength = 10.59), activation (bust strength = 9.63), and motor recovery (bust strength = 8.92). Over time, positron emission tomography (1999–2011), unilateral neglect (1999–2010), activation (2000–2010), amphetamine (2000–2010), and induced movement therapy (2005–2014) have received the most sustained attention for these keywords. Participation (2019–2020), experience (2020–2022), and connectivity (2020–2022) have been used more recently and may become hot research topics in the future.

## 4. Discussion

### 4.1. Global trends of post-stroke aphasia research

In this study, a bibliometric visualization analysis of 2,325 publications on post-stroke aphasia rehabilitation from 1998 to 2022 was conducted to demonstrate the ongoing scholarly work in this field. The findings revealed a trend of yearly growth in the number of publications, with a particularly pronounced trend of growth from 2019 to 2021. The highest number of publications was observed in 2021 (232 articles), and the results suggest that research on post-stroke aphasia rehabilitation is receiving increasing attention from countries around the world.

The University of Queensland has the highest number of publications and citations (137 articles, 3,771 citations), and one researcher from this institution—Worrall L—is the author of the most articles in the field of post-stroke aphasia rehabilitation (51 articles). This finding is strongly supported by the excellent academic environment and research base. In addition, the collaborative network of institutions shows that interinstitutional collaboration is mainly distributed in Europe and the United States, which may be related to the good national economic situation and high investment in health care in developed countries. On the other hand, Asian countries are absent from the top 10 institutions in post-stroke aphasia rehabilitation research, which may hinder the development of the field. The authors’ network of collaborative clusters shows that collaborative exchanges between authors within clusters are more extensive, while links between different clusters are still relatively lacking. In the future, international research collaboration should be expanded even more to advance the advancement of the discipline and the standard of research as a whole.

Articles on post-stroke aphasia rehabilitation covered several fields, with clinical neurology (882 articles), rehabilitation (877 articles), and neuroscience (745 articles) being the most popular and peripheral vascular disease having the highest number of single citations (66.97 per article). Other areas of articles in this field include linguistics, audiology speech-language pathology, psychology, peripheral vascular disease, behavioral sciences, sport sciences, psychiatry, and neuroimaging, indicating that post-stroke aphasia rehabilitation is a complex problem that requires multidisciplinary intervention.

Peer-reviewed journals are important vehicles for academic publications and have the important responsibility of publishing the latest research findings. The top journals in this field include Aphasiology (254 articles, 4,800 citations), followed by Disability and Rehabilitation and Stroke. Aphasiology publishes articles on the pathophysiology, clinical, psychological, linguistic, and social sciences of aphasia and greatly facilitates interdisciplinary communication around the field. Stroke has the highest impact factor (IF = 10.2) among the top 10 journals and was founded by the American Heart Association to provide a platform for high-impact stroke research from an international perspective. In addition, three journals are devoted to the scope of physical rehabilitation treatment for post-stroke aphasia: Disability and Rehabilitation, Topics in Stroke Rehabilitation, and Archives of Physical Medicine and Rehabilitation. Brain and Language and Neurorehabilitation and Neural Repair are dedicated to the neurobiology and neurocognitive basis of post-stroke aphasia, while Neuropsychological Rehabilitation focuses on the neurorehabilitation of post-stroke aphasia and related neuropsychological assessment. Neuropsychological Rehabilitation focuses on neurorehabilitation and related neuropsychological assessment of post-stroke aphasia. By analyzing the dual-map overlaps of the posting journals to understand the citation trajectories between fields, it can be seen that post-stroke aphasia rehabilitation is influenced by fields ranging from molecular biology, neuromotor, and medical to psychological, economic, and social. This shows that post-stroke aphasia rehabilitation is multidisciplinary in nature, and future research could consider the dynamics of journals in these fields.

### 4.2. Research hotspots and frontiers

Through reference co-citation and keyword co-occurrence analysis, the research focus and development trend in the field of post-stroke aphasia rehabilitation can be identified. The research hotspots and frontiers of the field can then be identified by combining temporal overlap visualization and burst detection of keywords. This will help form an overall understanding of the research progress and future directions of post-stroke aphasia rehabilitation. The potential future research directions are as follows.

#### 4.2.1. Plasticity mechanisms of neurolinguistics networks in post-stroke aphasia

Current research suggests that after stroke-induced brain lesions, the neurofunctional network of the brain can be reorganized to help compensate for and partially recover areas of the damaged neural network (Joy and Carmichael, 2021). Brain function is reorganized in three consecutive phases: acute, subacute, and chronic, the first two being spontaneous neuroplastic changes, while the chronic phase requires the provision of various specific interventions to promote further functional improvement (Rossini and Forno, 2004; Kerr et al., 2011). Factors affecting neuroplasticity in post-stroke aphasia include intrinsic factors (e.g., the age of onset, blood perfusion, stroke lesion characteristics and language impairment patterns) and extrinsic environmental factors (e.g., treatment and psychosocial variables) (Hartwigsen and Saur, 2019). Functional neuroimaging can reveal the neural mechanisms of language network reorganization by mapping neural activity and functional and structural connectivity changes to predict outcomes and improve treatment efficiency (Hartwigsen and Saur, 2019). Among numerous methods, functional magnetic resonance imaging (fMRI) has been most often used to examine functional connectivity changes in networks associated with language recovery. By recording changes in connectivity over time in the absence of a task (e.g., resting state), fMRI can explore the natural processes and patterns of language function recovery after brain injury; comparison with task-based connectivity changes allows the construction of a model for treatment-induced language function recovery (Stockert et al., 2020; Wilson and Schneck, 2020). Electrophysiological methods are also frequently used to study neural changes in patients with post-stroke aphasia. Magnetoencephalography (MEG) and electroencephalography (EEG) are the most commonly used measurement tools (Johnson and Fridriksson, 2022). By tracing the spatial topography of specific oscillatory bands and frequencies associated with preserved language abilities after stroke, electrophysiology can be used to explore the electromagnetic reorganization activity of communication connections between electrophysiological regions in post-stroke aphasia (Shah-Basak et al., 2022). In addition, research on stroke recovery biomarkers (SRBs) is booming, while SRBs that promote language reorganization are still poorly understood and could be incorporated into post-stroke aphasia rehabilitation in the future (Boyd et al., 2017; Ulanov and Shtyrov, 2022). In the future, multivariate analysis, using multiple functional neuroimaging and non-invasive brain stimulation approaches, can be adopted to integrate and analyze the collected data to more acutely capture the neural network changes in the brain that drive recovery from post-stroke aphasia (Hartwigsen and Saur, 2019). In addition, further linking speech therapy to neural changes in specific lesion sites of language activity and connectivity, and actively explore and develop treatment-induced neural reorganization models that can provide the basis for personalized targeted rehabilitation of post-stroke aphasia (Mattioli, 2019).

#### 4.2.2. Assessment of language function in post-stroke aphasia

A large stroke study found that it is practically feasible to follow a scientific framework for selecting and implementing standardized language assessment procedures in acute care and inpatient rehabilitation settings to assess early changes in language performance at multiple time points (Stipancic et al., 2019; Schliep et al., 2020). In practice, Language function assessment provides clinicians with information for the differential diagnosis of aphasia, measures the effectiveness of various treatment modalities, and provides clues for investigating the anatomical and biological correlates of language function, as well as the location and function of damaged brain structures (Kertesz, 2022). Numerous previous studies have also extensively investigated the physical and psychological influences on post-stroke aphasia using language function assessment tools and have longitudinally followed the long-term recovery of language function in patients with post-stroke aphasic patients to monitor their language improvement over time and to guide individualized speech therapy (Wang et al., 2018; Stipancic et al., 2019). Since the scoring of language function and sign manipulation usually require professional guidance, meeting the needs of aphasic patients for teleconsultation has become a hot topic. A videoconferencing management approach for WAB-R that allows language and communication assessments to be operated remotely through videoconferencing technologies (VCT) and can serve as a viable alternative to face-to-face assessments (Dekhtyar et al., 2020). In addition, various language function screening tools such as the Language Screening Test (LAST) (Flamand-Roze et al., 2011), ScreeLing (Doesborgh et al., 2003), etc., have been developed to enable treatment-oriented assessment of language function in a short period of time in a clinical setting (El Hachioui et al., 2017). Among them, The Short and Tailored Evaluation of Language Ability (STELA), a newly developed computer-based Japanese language proficiency assessment system for patients with aphasia, simplifies the administration of test entries by computer, automates the recording of scores and supplements them with additional objective measurements (e.g., reaction time), greatly reducing the testing time for patients (Inamoto et al., 2023). Since most current tools for assessing language function follow neural models rather than psycholinguistic theories, the assessment validity of post-stroke aphasia patients may be affected if they also have cognitive impairment or psycho-behavioral disorders, and other coexisting neurological deficits, such as apraxia or visual field deficits, may hinder the prognostic outcome of post-stroke aphasic patients and need to be evaluated together (Kertesz, 2022). At the same time, there are also limitations in current language function assessment methods, such as linguistic differences between cultures and geographic regions, and whether the localized scales have reliability and validity, which can affect the assessment of language function and its efficacy (Sun et al., 2020). It follows that more language function assessment tools need to be developed and validated in the future to explore the feasibility of their measurement in different countries and populations. In addition, the use of intelligent medical assessment methods will be further expanded in the future for different populations and in a wider range of clinical settings, providing the prerequisites for conducting extensive clinical practice with quantitative or automated instruments for post-stroke aphasia assessment by increasing clinical practice based on this aspect.

#### 4.2.3. Language rehabilitation modalities for post-stroke aphasia

Speech and language therapy (SLT) is by far the most popular method for treating post-stroke aphasia. “Speech and language therapy for aphasia following stroke” is the most frequently cited reference for this article (102 articles) (Brady et al., 2016). This systematic review reports that SLT improves reading, writing, communication function, and speech expression in post-stroke aphasic patients and suggests that high-intensity training improves language skills, but there is often a high rate of disengagement. A multicenter, single-blinded, randomized controlled trial found that self-managed, computerized speech and language therapy (CSLT) plus usual care significantly improved word finding in patients with chronic stroke aphasia and that patients improved their participation in recovery through the use of self-managed CSLT (Palmer et al., 2019). Although there is evidence that people with post-stroke aphasia can benefit from SLT, transportation closures, time conflicts, or lack of appropriate rehabilitation services in hospitals make long-term recovery very difficult (Morris et al., 2017). Therefore, it is important to ensure proper treatment dosage and to maintain long-term recovery. Research indicates that tele-rehabilitation for aphasia is not only has equal validity compared to traditional face-to-face language rehabilitation, but also allows patients to enjoy high-quality rehabilitation in a familiar environment that is more convenient (Cacciante et al., 2021). Currently, remote rehabilitation via videoconferencing is considered a viable rehabilitation model for post-stroke aphasia (Øra et al., 2020), through web-based oral reading of language for aphasia (Web ORLA), people with chronic aphasia are encouraged to perform repetitive choral and independent readings with a virtual therapist, which can lead to improvements in language function (Cherney et al., 2021). In addition, it has been suggested that the virtual context provided by VR technology can promote the ecological effectiveness of speech therapy (Bu et al., 2022). It is thus clear that providing precise rehabilitation goals for post-stroke aphasic patients and exploring rehabilitation tools that are motivating and not easily limited by time and place will become a hot topic in this field. In terms of physiotherapy, non-invasive brain stimulation, such as transcranial direct current stimulation (tDCS), can improve post-stroke aphasia by modulating cortical excitability as an adjunct to synergistic SLT (Lefaucheur et al., 2017; Hartwigsen and Saur, 2019). However, most of the current studies have small sample sizes, inconsistent intervention durations, and significant differences between interventions and participants across studies. Large, multicenter randomized controlled trials are still needed in the future to clarify the optimal time to start recovery from post-stroke aphasia, the type of aphasia, and the stimulation parameters of physical therapy (Picano et al., 2021; REhabilitation and recovery of peopLE with Aphasia after StrokE (Release) Collaborators, 2022). When assessing outcomes, the impact of the intervention on social communication or quality of life should be included in addition to attention to outcome indicators at the site of injury.

#### 4.2.4. Rehabilitation needs and engagement experiences of post-stroke aphasic patients

Communication barriers often limit the ability of people with post-stroke aphasia to communicate with others, preventing them from accessing appropriate rehabilitation information and services (Worrall et al., 2011). Family support, acquaintance with others, and a supportive social environment can improve the participation in rehabilitation and quality of life of people with post-stroke aphasia, and accessible information and collaborative interaction with health professionals can lead to self-rehabilitation management of people with post-stroke aphasia (Manning et al., 2019; Manning et al., 2020). A meta-analysis examined the adjustment process of family communication patterns during post-stroke aphasia and the factors that facilitate and hinder family communication, encouraging an open approach to family communication and a variety of modern technologies to support people with post-stroke aphasia and family members (Ramazanu et al., 2022). Qualitative findings from the perspective of patients with aphasia (Nichol et al., 2022) indicate that self-management of aphasia involves skills in daily conversation, social interaction, and life participation. Community groups and communication partners can provide peer support and assistance with self-management, health professionals can provide information and educational training in related areas, and people with aphasia can learn technology for communication and daily interaction. Current qualitative research approaches to explore in depth the rehabilitation needs and engagement experiences of people with post-stroke aphasia are topical. However, it is worth noting that post-stroke aphasia also has an important impact on the lives of family members (Grawburg et al., 2019), and further investigation of the rehabilitation goals of family members of patients with post-stroke aphasia is needed (Zawawi et al., 2020). In addition, further adoption of a mixed study approach will help us to better personalize rehabilitation for patients with post-stroke aphasia (Devanga et al., 2021).

## 5. Strengths and limitations

This study established a visual literature network based on bibliometric and visual analysis to review the progress and trends of scientific research on post-stroke aphasia rehabilitation worldwide. This paper makes it simple for researchers to understand the status, hotspots, and trends of current research in this area. However, this work has some limitations. First, due to software limitations, we only analyzed publications from WoSCC; thus, some important studies may be excluded, and it would be useful to combine the results of this study with those from other databases such as Scopus and PubMed. Second, because this study only included articles and reviews from English-language publications, it is possible that other significant literature was omitted. Additionally, some recently published high-quality papers were not given much weight by this study because of their recent publication and low number of citations, which could lead to differences between the results and reality.

## 6. Conclusion

This study provided the first bibliometric visualization analysis of post-stroke aphasia rehabilitation research. The Web of Science database, VOSviewer, and CiteSpace software were used to provide a more scientific and intuitive overview of research in this field. In addition, we presented the lineage of the development of post-stroke aphasia rehabilitation, as well as the main trends, research hotspots, and frontiers. The visualization analysis shows that the research in this field is rapidly developing, and relevant literature is emerging. Currently, the frontiers and hot spots for future research include plasticity mechanisms of neurolinguistics networks, language function assessment, language rehabilitation modalities, and patients’ rehabilitation needs and participation experiences for post-stroke aphasia. In conclusion, this study’s findings can serve as a useful guide for researchers interested in post-stroke aphasia rehabilitation as well as a source of information and ideas for further related studies.

## Data availability statement

The original contributions presented in this study are included in the article/supplementary material, further inquiries can be directed to the corresponding authors.

## Author contributions

HW and DZ: research concept and design. HW and ZC: collection and assembly of data and writing the manuscript. HW, ZC, and CL: data analysis and interpretation. SL, JZ, and YX: critical revision of the manuscript. CL, DZ, and YH: final approval of the manuscript. All authors read and approved the manuscript.

## References

[B1] Baker C. Worrall L. Rose M. Ryan B. (2020). ‘It was really dark’: The experiences and preferences of people with aphasia to manage mood changes and depression. *Aphasiology* 34 19–46. 10.1080/02687038.2019.1673304

[B2] Baker C. Worrall L. Rose M. Hudson K. Ryan B. O’Byrne L. (2018). A systematic review of rehabilitation interventions to prevent and treat depression in post-stroke aphasia. *Disabil. Rehabil.* 40 1870–1892. 10.1080/09638288.2017.1315181 28420284

[B3] Baker J. M. Rorden C. Fridriksson J. (2010). Using transcranial direct-current stimulation to treat stroke patients with aphasia. *Stroke* 41 1229–1236. 10.1161/STROKEAHA.109.576785 20395612 PMC2876210

[B4] Boyd L. A. Hayward K. S. Ward N. S. Stinear C. M. Rosso C. Fisher R. J. (2017). Biomarkers of stroke recovery: Consensus-based core recommendations from the stroke recovery and rehabilitation roundtable. *Neurorehabil. Neural Repair.* 31 864–876. 10.1177/1545968317732680 29233071

[B5] Brady M. C. Kelly H. Godwin J. Enderby P. Campbell P. (2016). Speech and language therapy for aphasia following stroke. *Cochrane Database Syst. Rev.* 2016:CD000425. 10.1002/14651858.CD000425.pub4 27245310 PMC8078645

[B6] Breitenstein C. Grewe T. Flöel A. Ziegler W. Springer L. Martus P. (2017). Intensive speech and language therapy in patients with chronic aphasia after stroke: A randomised, open-label, blinded-endpoint, controlled trial in a health-care setting. *Lancet* 389 1528–1538. 10.1016/S0140-6736(17)30067-3 28256356

[B7] Bu X. Ng P. H. Tong Y. Chen P. Q. Fan R. Tang Q. (2022). A mobile-based virtual reality speech rehabilitation app for patients with aphasia after stroke: Development and pilot usability study. *JMIR Serious Games* 10:e30196. 10.2196/30196 35389349 PMC9031062

[B8] Cacciante L. Kiper P. Garzon M. Baldan F. Federico S. Turolla A. (2021). Telerehabilitation for people with aphasia: A systematic review and meta-analysis. *J. Commun. Disord.* 92:106111. 10.1016/j.jcomdis.2021.106111 34052617

[B9] Chen C. (2004). Searching for intellectual turning points: Progressive knowledge domain visualization. *Proc. Natl. Acad. Sci. U.S.A.* 101 5303–5310. 10.1073/pnas.0307513100 14724295 PMC387312

[B10] Chen C. (2006). CiteSpace II: Detecting and visualizing emerging trends and transient patterns in scientific literature. *J. Am. Soc. inform. Sci. Technol.* 57 359–377. 10.1002/ASI.20317

[B11] Chen C. (2014). The citespace manual. *Coll. Comput. Inform.* 1 1–84.

[B12] Chen C. (2017). Science mapping: A systematic review of the literature. *J. Data Inform. Sci.* 2 1–40. 10.1515/jdis-2017-0006

[B13] Chen C. Chen Y. Horowitz M. Hou H. Liu Z. Pellegrino D. (2009). Towards an explanatory and computational theory of scientific discovery. *J. Inform.* 3 191–209. 10.1016/j.joi.2009.03.004

[B14] Chen C. Ibekwe-SanJuan F. Hou J. (2010). The structure and dynamics of cocitation clusters: A multiple-perspective cocitation analysis. *J. Am. Soc. inform. Sci. Technol.* 61 1386–1409. 10.1002/asi.21309

[B15] Cherney L. R. Lee J. B. Kim K.-Y. A. van Vuuren S. (2021). Web-based oral reading for language in aphasia (Web ORLA^®^): A pilot randomized control trial. *Clin. Rehabil.* 35 976–987. 10.1177/0269215520988475 33472420

[B16] Dalemans R. J. De Witte L. Wade D. van den Heuvel W. (2010). Social participation through the eyes of people with aphasia. *Int. J. Lang. Commun. Disord.* 45 537–550. 10.3109/13682820903223633 19839875

[B17] Dekhtyar M. Braun E. J. Billot A. Foo L. Kiran S. (2020). Videoconference administration of the western aphasia battery–revised: Feasibility and validity. *Am. J. Speech-Lang. Pathol.* 29 673–687. 10.1044/2019_AJSLP-19-00023 32191122 PMC7842871

[B18] Devanga S. R. Sherrill M. Hengst J. A. (2021). The efficacy of collaborative referencing intervention in chronic aphasia: A mixed-methods study. *Am. J. Speech-Lang. Pathol.* 30 407–424. 10.1044/2020_AJSLP-19-00108 32585113

[B19] Doesborgh S. J. van de Sandt-Koenderman W. M. Dippel D. W. van Harskamp F. Koudstaal P. J. Visch-Brink E. G. (2003). Linguistic deficits in the acute phase of stroke. *J. Neurol.* 250 977–982. 10.1007/s00415-003-1134-9 12928919

[B20] El Hachioui H. Visch-Brink E. G. de Lau L. M. van de Sandt-Koenderman M. W. Nouwens F. Koudstaal P. J. (2017). Screening tests for aphasia in patients with stroke: A systematic review. *J. Neurol.* 264 211–220. 10.1007/s00415-016-8170-8 27260296 PMC5306063

[B21] Ellegaard O. Wallin J. A. (2015). The bibliometric analysis of scholarly production: How great is the impact? *Scientometrics* 105 1809–1831. 10.1007/s11192-015-1645-z 26594073 PMC4643120

[B22] Feigin V. L. Stark B. A. Johnson C. O. Roth G. A. Bisignano C. Abady G. G. (2021). Global, regional, and national burden of stroke and its risk factors, 1990–2019: A systematic analysis for the Global Burden of Disease Study 2019. *Lancet Neurol.* 20 795–820. 10.1016/S1474-4422(21)00252-0 34487721 PMC8443449

[B23] Flamand-Roze C. Falissard B. Roze E. Maintigneux L. Beziz J. Chacon A. (2011). Validation of a new language screening tool for patients with acute stroke: The Language Screening Test (LAST). *Stroke* 42 1224–1229. 10.1161/strokeaha.110.609503 21487118

[B24] Floel A. Cohen L. G. (2010). Recovery of function in humans: Cortical stimulation and pharmacological treatments after stroke. *Neurobiol. Dis.* 37 243–251. 10.1016/j.nbd.2009.05.027 19520165 PMC4886709

[B25] Flowers H. L. Skoretz S. A. Silver F. L. Rochon E. Fang J. Flamand-Roze C. (2016). Poststroke aphasia frequency, recovery, and outcomes: A systematic review and meta-analysis. *Arch. Phys. Med. Rehabil.* 97 2188–2201.e8. 10.1016/j.apmr.2016.03.006 27063364

[B26] Fridriksson J. Richardson J. D. Baker J. M. Rorden C. (2011). Transcranial direct current stimulation improves naming reaction time in fluent aphasia: A double-blind, sham-controlled study. *Stroke* 42 819–821. 10.1161/strokeaha.110.600288 21233468 PMC8210639

[B27] Fridriksson J. Rorden C. Elm J. Sen S. George M. S. Bonilha L. (2018). Transcranial direct current stimulation vs sham stimulation to treat aphasia after stroke: A randomized clinical trial. *JAMA Neurol.* 75 1470–1476. 10.1001/jamaneurol.2018.2287 30128538 PMC6583191

[B28] Grawburg M. Howe T. Worrall L. Scarinci N. (2019). Family-centered care in aphasia: Assessment of third-party disability in family members with the family aphasia measure of life impact. *Top. Lang. Disord.* 39 29–54. 10.1097/TLD.0000000000000176

[B29] Hamilton R. H. Chrysikou E. G. Coslett B. (2011). Mechanisms of aphasia recovery after stroke and the role of noninvasive brain stimulation. *Brain Lang.* 118 40–50. 10.1016/j.bandl.2011.02.005 21459427 PMC3109088

[B30] Hartwigsen G. Saur D. (2019). Neuroimaging of stroke recovery from aphasia - Insights into plasticity of the human language network. *Neuroimage* 190 14–31. 10.1016/j.neuroimage.2017.11.056 29175498

[B31] Inamoto Y. Mukaino M. Imaeda S. Sawada M. Satoji K. Nagai A. (2023). a tablet-based aphasia assessment system “STELA”: Feasibility and validation study. *JMIR Form. Res.* 7:e42219. 10.2196/42219 36753308 PMC9947769

[B32] Johnson L. P. Fridriksson J. (2022). Electrophysiologic evidence of reorganization in poststroke aphasia. *Handb. Clin. Neurol.* 185 167–174. 10.1016/b978-0-12-823384-9.00020-7 35078597

[B33] Joy M. T. Carmichael S. T. (2021). Encouraging an excitable brain state: Mechanisms of brain repair in stroke. *Nat. Rev. Neurosci.* 22 38–53. 10.1038/s41583-020-00396-7 33184469 PMC10625167

[B34] Kerr A. L. Cheng S. Y. Jones T. A. (2011). Experience-dependent neural plasticity in the adult damaged brain. *J. Commun. Disord.* 44 538–548. 10.1016/j.jcomdis.2011.04.011 21620413 PMC3162127

[B35] Kertesz A. (1982). *Western aphasia battery test manual.* New York, NY: Psychological Corporation.

[B36] Kertesz A. (2007). *Western Aphasia Battery(Revised).* San Antonio, TX: PsychCorp.

[B37] Kertesz A. (2022). The western aphasia battery: A systematic review of research and clinical applications. *Aphasiology* 36 21–50. 10.1080/02687038.2020.1852002

[B38] Kiran S. Thompson C. K. (2019). Neuroplasticity of language networks in aphasia: Advances, updates, and future challenges. *Front. Neurol.* 10:295. 10.3389/fneur.2019.00295 31001187 PMC6454116

[B39] Lefaucheur J.-P. Antal A. Ayache S. S. Benninger D. H. Brunelin J. Cogiamanian F. (2017). Evidence-based guidelines on the therapeutic use of transcranial direct current stimulation (tDCS). *Clin. Neurophysiol.* 128 56–92. 10.1016/j.clinph.2016.10.087 27866120

[B40] Li C. Shu X. Liu X. (2022). Research hotspots and frontiers in post stroke pain: A bibliometric analysis study. *Front. Mol. Neurosci.* 15:905679. 10.3389/fnmol.2022.905679 35645732 PMC9137410

[B41] Manning M. Cuskelly C. Russ E. Franklin S. (2020). Supporting people with post-stroke aphasia to live well: A cross-sectional survey of speech & language therapists in Ireland. *Health Soc. Care Community* 28 2105–2116. 10.1111/hsc.13021 32462685

[B42] Manning M. MacFarlane A. Hickey A. Franklin S. (2019). Perspectives of people with aphasia post-stroke towards personal recovery and living successfully: A systematic review and thematic synthesis. *PLoS One* 14:e0214200. 10.1371/journal.pone.0214200 30901359 PMC6430359

[B43] Mattioli F. (2019). The clinical management and rehabilitation of post stroke aphasia in Italy: Evidences from the literature and clinical experience. *Neurol. Sci.* 40 1329–1334. 10.1007/s10072-019-03844-0 30900098

[B44] Morris J. H. Oliver T. Kroll T. Joice S. Williams B. (2017). Physical activity participation in community dwelling stroke survivors: Synergy and dissonance between motivation and capability. A qualitative study. *Physiotherapy* 103 311–321. 10.1016/j.physio.2016.05.001 27613082

[B45] Newman M. E. (2006). Modularity and community structure in networks. *Proc. Natl. Acad. Sci. U.S.A.* 103 8577–8582. 16723398 10.1073/pnas.0601602103 PMC1482622

[B46] Nichol L. Wallace S. J. Pitt R. Rodriguez A. D. Diong Z. Z. Hill A. J. (2022). People with aphasia share their views on self-management and the role of technology to support self-management of aphasia. *Disabil. Rehabil.* 44 7399–7412. 10.1080/09638288.2021.1989501 34657536

[B47] Øra H. P. Kirmess M. Brady M. C. Partee I. Hognestad R. B. Johannessen B. B. (2020). The effect of augmented speech-language therapy delivered by telerehabilitation on poststroke aphasia—a pilot randomized controlled trial. *Clin. Rehabil.* 34 369–381. 10.1177/0269215519896616 31903800

[B48] Palmer R. Dimairo M. Cooper C. Enderby P. Brady M. Bowen A. (2019). Self-managed, computerised speech and language therapy for patients with chronic aphasia post-stroke compared with usual care or attention control (Big CACTUS): A multicentre, single-blinded, randomised controlled trial. *Lancet Neurol.* 18 821–833. 10.1016/s1474-4422(19)30192-9 31397288 PMC6700375

[B49] Picano C. Quadrini A. Pisano F. Marangolo P. (2021). Adjunctive approaches to aphasia rehabilitation: A review on efficacy and safety. *Brain Sci.* 11:41. 10.3390/brainsci11010041 33401678 PMC7823462

[B50] Ramazanu S. Chisale M. R. Baby P. Wu V. X. Mbakaya B. C. (2022). Meta-synthesis of family communication patterns during post-stroke vascular aphasia: Evidence to guide practice. *Worldviews Evid.-Based Nurs.* 19 282–296. 10.1111/wvn.12580 35587739

[B51] REhabilitation and recovery of peopLE with Aphasia after StrokE (Release) Collaborators (2022). Dosage, intensity, and frequency of language therapy for aphasia: A systematic review–based, individual participant data network meta-analysis. *Stroke* 53 956–967. 10.1161/STROKEAHA.121.035216 34847708 PMC8884127

[B52] Rossini P. M. Forno G. D. (2004). Neuronal post-stroke plasticity in the adult. *Restorat. Neurol. Neurosci.* 22 193–206. 15502265

[B53] Rousseeuw P. J. (1987). Silhouettes: A graphical aid to the interpretation and validation of cluster analysis. *J. Comput. Appl. Math.* 20 53–65.

[B54] Sabe M. Chen C. Perez N. Solmi M. Mucci A. Galderisi S. (2023). Thirty years of research on negative symptoms of schizophrenia: A scientometric analysis of hotspots, bursts, and research trends. *Neurosci. Biobehav. Rev.* 144:104979. 10.1016/j.neubiorev.2022.104979 36463972

[B55] Saur D. Lange R. Baumgaertner A. Schraknepper V. Willmes K. Rijntjes M. (2006). Dynamics of language reorganization after stroke. *Brain* 129 1371–1384. 10.1093/brain/awl090 16638796

[B56] Schliep M. E. Kasparian L. Kaminski O. Tierney-Hendricks C. Ayuk E. Brady Wagner L. (2020). Implementing a standardized language evaluation in the acute phases of aphasia: Linking evidence-based practice and practice-based evidence. *Front. Neurol.* 11:412. 10.3389/fneur.2020.00412 32547472 PMC7278284

[B57] Shah-Basak P. Sivaratnam G. Teti S. Deschamps T. Kielar A. Jokel R. (2022). Electrophysiological connectivity markers of preserved language functions in post-stroke aphasia. *Neuroimage Clin.* 34:103036. 10.1016/j.nicl.2022.103036 35561556 PMC9111985

[B58] Stipancic K. L. Borders J. C. Brates D. Thibeault S. L. (2019). Prospective investigation of incidence and co-occurrence of dysphagia, dysarthria, and aphasia following ischemic stroke. *Am. J. Speech Lang. Pathol.* 28 188–194. 10.1044/2018_ajslp-18-0136 31072162

[B59] Stockert A. Wawrzyniak M. Klingbeil J. Wrede K. Kümmerer D. Hartwigsen G. (2020). Dynamics of language reorganization after left temporo-parietal and frontal stroke. *Brain* 143 844–861. 10.1093/brain/awaa023 32068789

[B60] Sun M. Zhan Z. Chen B. Xin J. Chen X. Yu E. (2020). Development and application of a chinese version of the language screening test (CLAST) in post-stroke patients. *Medicine* 99:e22165. 10.1097/md.0000000000022165 32925781 PMC7489636

[B61] Synnestvedt M. B. Chen C. Holmes J. H. (2005). CiteSpace II: Visualization and knowledge discovery in bibliographic databases. *AMIA Annu. Symp. Proc.* 2005 724–728. 16779135 PMC1560567

[B62] Ulanov M. Shtyrov Y. (2022). Oscillatory beta/alpha band modulations: A potential biomarker of functional language and motor recovery in chronic stroke? *Front. Hum. Neurosci.* 16:940845. 10.3389/fnhum.2022.940845 36226263 PMC9549964

[B63] Van Eck N. Waltman L. (2010). Software survey: VOSviewer, a computer program for bibliometric mapping. *Scientometrics* 84 523–538. 10.1007/s11192-009-0146-3 20585380 PMC2883932

[B64] Wang S. Wang C. X. Zhang N. Xiang Y. T. Yang Y. Shi Y. Z. (2018). The association between post-stroke depression, aphasia, and physical independence in stroke patients at 3-month follow-up. *Front. Psychiatry* 9:374. 10.3389/fpsyt.2018.00374 30177891 PMC6110154

[B65] Wilson S. M. Schneck S. M. (2020). Neuroplasticity in post-stroke aphasia: A systematic review and meta-analysis of functional imaging studies of reorganization of language processing. *Neurobiol. Lang.* 2 22–82. 10.1162/nol_a_00025 33884373 PMC8057712

[B66] Worrall L. Sherratt S. Rogers P. Howe T. Hersh D. Ferguson A. (2011). What people with aphasia want: Their goals according to the ICF. *Aphasiology* 25 309–322. 10.1080/02687038.2010.508530

[B67] Wray F. Clarke D. (2017). Longer-term needs of stroke survivors with communication difficulties living in the community: A systematic review and thematic synthesis of qualitative studies. *BMJ Open* 7:e017944. 10.1136/bmjopen-2017-017944 PMC5640038 28988185

[B68] Zawawi N. Aziz N. Fisher R. Ahmad K. Walker M. F. (2020). The unmet needs of stroke survivors and stroke caregivers: A systematic narrative review. *J. Stroke Cerebrovasc. Dis.* 29:104875. 10.1016/j.jstrokecerebrovasdis.2020.104875 32689648

